# The effect of dosing sequence of programmed death receptor-1 inhibitors combined with chemotherapy on adverse effects in the real world

**DOI:** 10.3389/fonc.2025.1645091

**Published:** 2025-12-15

**Authors:** HaoChun Tang, Ziyuan Zhou, Yuelin Zhang, Jun Pan, Jun Meng

**Affiliations:** 1 Department of Pharmacy, National Cancer Center/National Clinical Research Center for Cancer/Cancer Hospital and Shenzhen Hospital, Chinese Academic of Medical Sciences and Peking Union Medical College, Shenzhen, China; 2 School of Pharmacy, Guangdong Medical University, Dongguan, Guangdong, China; 3 Shenzhen Hospital, Peking University, Shenzhen, Guangdong, China

**Keywords:** programmed cell death protein-1 inhibitor, chemotherapy, combined dosing, dosing sequence, immune-related adverse effects

## Abstract

**Objective:**

This study aims to explore the impact of different dosing sequences on the occurrence of adverse events (AEs) when PD-1 inhibitors are combined with chemotherapy, so as to provide a basis for optimising clinical medication regimens.

**Methods:**

Retrospective cohort study and retrospective analysis were conducted on 400 cases of tumour patients who received PD-1 inhibitors combined with chemotherapy in a specialised oncology hospital from January to December 2024. They were divided into two groups according to the dosing sequence: 200 cases in the post-chemotherapy group (PD-1 inhibitors administered first followed by chemotherapy) and 200 cases in the pre-chemotherapy group (chemotherapy administered first followed by PD-1 inhibitors). The baseline characteristics, incidence, types and grades of adverse events in the two groups were counted, and the differences between the groups were compared.

**Results:**

Among the 400 patients, males accounted for 63.5%, people aged 40–69 accounted for 72.5%, and the main diseases were lung cancer (28.5%), hepatocellular carcinoma (15.0%) and gastric cancer (10.5%). The total incidence of adverse events was 65.0%, among which the incidence of adverse events in the pre-chemotherapy group (75.0%) was significantly higher than that in the post-chemotherapy group, and the difference was statistically significant (P<0.05). The main types of adverse events were haematological toxicity (22.25%), hepatotoxicity (21.25%) and dermatotoxicity (13.5%). There was no statistically significant difference in the incidence of the same type of adverse events between the two groups (P>0.05). The toxicity grading was mainly G1-G2 (316 cases, 79.0%), and G3 and above accounted for 11.5%. There was no significant difference in the grading distribution between the two groups (P>0.05).

**Conclusion:**

The dosing sequence of PD-1 inhibitors combined with chemotherapy is closely related to the occurrence of adverse events. Administering PD-1 inhibitors first can reduce the risk of adverse events, and the choice of dosing sequence should be paid attention to in clinical practice.

## 1 Introduction

At present, programmed cell death receptor-1 (PD-1) signalling pathway has become a target of tumour immunotherapy, which is an important direction of immunotherapy in recent years (1). In order to improve antitumour efficacy and delay drug resistance, combination therapy has become a mainstream trend (2). Combination drugs may have an impact on efficacy and toxicity due to drug-drug interactions or cycle-specificity of drug action, and thus generally have a fixed order of administration (3). In immunotherapy combination chemotherapy regimens, PD-1 inhibitors can enhance immune activity and inhibit the binding of PD-1 to PD-L1, and the immune cells activated non-specifically will attack normal cells while fighting against tumour cells, which will lead to immune-related adverse events in the body, which are mostly manifested as endocrine disorders, skin toxicity and gastrointestinal tract reactions etc. (4, 5). Although the four PD-1 inhibitors, pabolizumab, tirilizumab, sindilizumab and karelizumab, as mention in the drug insert, PD-1 inhibitor must be used before the chemotherapeutic agent, most clinicians in the real world do not strictly follow the order of administration required by the insert.

In this paper, we used retrospective analysis to investigate the occurrence of AEs in patients treated with PD-1 inhibitor combination chemotherapy regimens in a hospital in 1˜December 2024, to explore the effect of dosing order on AEs, and to provide clues for optimising the best dosing regimen by adjusting the order of dosing when immunotherapy is combined with chemotherapy, to reduce adverse events, and to promote the rational use of medication in clinics.

## 2 Information and methods

### 2.1 Sources of cases

A retrospective analysis was used to collect PD-1 inhibitors used in a specialised oncology hospital in January˜December 2024, and a total of seven PD-1 inhibitors were involved in the study, namely, pabolizumab injection (Hangzhou Merck Sharp & Dohme Pharmaceuticals Co., Ltd, specification: 4 ml/100 mg, batch no.: X007115), nabulizumab injection (Bristol-Myers Squibb Pharma EEIG, specification: 4ml/40mg, 10ml/100mg, batch number: ACH6876, ACF3167), Sindelizumab Injection (Xindal Biopharmaceutical Co., Ltd., specification: 10ml:100mg, batch number: DP2307004), Teraplizumab Injection (Ltd, specification: 6ml/240mg, batch no.: 202309058A), tirilizumab injection (Bazishenshu Shanghai Biotechnology Co., Ltd, specification: 10ml/100mg, batch no.: G202304112), carilizumab for injection (Suzhou Shengdia Bio-pharmaceutical Co., Ltd, specification: 200mg/vial, batch number: 202312033F) and srulizumab injection (Shanghai Fuhong Hanlin Bio-Pharmaceutical Co., Ltd, specification: 100mg/vial, batch number: 202312205) in combination with chemotherapy treatment for a total of 400 cases of tumour patients. Among them, 200 cases were treated with PD-1 inhibitor followed by chemotherapy (hereinafter referred to as the “post-chemotherapy group”), and 200 cases were treated with chemotherapy followed by PD-1 inhibitor (hereinafter referred to as the “pre-chemotherapy group”). It is worth noting that three other PD-1 inhibitors approved by the State Drug Administration of China (Separizumab, Putilizumab and Pavelvelizumab) were not yet used in clinical treatment during the study period.

### 2.2 Case entry criteria

#### 2.2.1 Inclusion criteria

(1) Patients diagnosed with malignant tumours, as there are many types of cancer involved, the judgement of the clinician who records the case shall prevail; (2) patients receiving at least one cycle of PD-1 inhibitor combination chemotherapy regimen; (3) a fixed order of medication; (4) complete results of laboratory tests such as blood routine, liver function and renal function before and after the treatment, and complete medical record data.

All patients have comparable physical condition, comorbidities, and previous treatments.

#### 2.2.2 Exclusion criteria

(1) Patients with immune-related underlying diseases, including post-transplantation patients; (2) women during pregnancy; (3) patients with non-small cell carcinoma who are positive for driver gene mutation; (4) patients who have received live vaccines during treatment.

### 2.3 Survey items

The general information (age, gender, diagnosis, department of consultation, etc.), medication information (name of drug, combined drug regimen, order of medication, etc.), and occurrence of AEs (signs and symptoms, toxicity grading, etc.) of the enrolled cases were accessed in the hospital information system (HIS), and the above information was recorded in Microsoft Excel software. Double checking was used to ensure the accuracy, authenticity and standardisation of the data.

### 2.4 AEs assessment criteria

Adverse events that were recorded in the medical records were collected and excluded those caused only by chemotherapy. Evaluate the association of adverse events with PD-1 inhibitors according to the relevant provisions in the “Management Methods for Reporting and Monitoring of Adverse Drug Reactions” (6) and exclude adverse events with an unlikely rating. Type rating and toxicity grading of AEs were performed using the “Management of adverse events and Toxicity of Immune Checkpoint Inhibitors in Oncology Therapy” (7) and the “Guidelines for the Management of Toxicity Associated with Immunocheckpoint Inhibitors (2024)” (8) issued by the CSCO as the criteria for reviewing the physician’s judgement of the adverse events.

The types of AEs included 14 types of skin toxicity, endocrine toxicity, cardiotoxicity, and malaise, and were classified into five grades based on toxicity: G1: mild toxicity; G2: moderate toxicity; G3: severe toxicity; G4: life-threatening toxicity; and G5: toxicity-related death, which corresponds to the Common Terminology Criteria for the Classification of Common Adverse Events (CTCAE_4.03), which was developed by the Cancer Research Institute of the National Institutes of Health (USA) for grading of adverse events.

### 2.5 Statistical analysis

SPSS software was applied for data processing, and the count data were expressed as the number of cases and percentages, and comparisons were made using the *χ*^2^test. It indicated that the difference was statistically significant.

## 3 Results

### 3.1 General conditions of patients

The data of 200 patients treated with PD-1 inhibitors combined with chemotherapy are shown in **Table 1**. The patients were predominantly male (63.5%), their ages were concentrated in the range of 40–69 years old (72.5%), and the diseases they suffered from were mainly lung cancer (28.5%), hepatocellular carcinoma (15.0%), and gastric cancer (10.5%). By calculating the p-values, the baseline table was generated. (**Table 2**) The data showed a statistically significant difference in the distribution of tumour types between the two groups (P < 0.001). For other variables (gender, age, PD-1 inhibitors), the p-values were all > 0.05, indicating no statistically significant differences.

**Table 1:** Basic information of patients treated with PD-1 inhibitors combined with chemotherapy.

Item	Sub-item	Post-chemotherapy group (number of cases)	Pre-chemotherapy group (number of cases)	Total (number of cases)	P value
Sex	Male	133	121	254	0.229
	Female	67	79	146	
Age (years)	Less than 30	4	8	12	0.831
	30-39	20	18	38	
	40-49	38	42	80	
	50-59	58	60	118	
	60-69	48	44	92	
	70-79	30	26	56	
	More than 80	2	2	4	
Tumour type	Lung cancer	86	28	114	<0.001
	Liver Cancer	12	48	60	
	Stomach cancer	12	30	42	
	Nasopharyngeal cancer	16	10	26	
	Lymphoma	14	8	22	
	Bowel Cancer	4	18	22	
	Oesophageal cancer	8	10	18	
	Cervical Cancer	14	2	16	
	Breast Cancer	4	10	14	
	Endometrial cancer	4	10	14	
	Gallbladder (duct) cancer	2	8	10	
	Malignant tumour of tongue	6	2	8	
	Kidney cancer	5	2	7	
	Malignant tumour of pharynx	4	2	6	
	Pancreatic cancer	0	5	5	
	Soft tissue sarcoma	0	5	5	
	Melanoma	4	0	4	
	Pleural mesothelioma	1	2	3	
	Ovarian cancer	2	0	2	
	Tumour of unknown primary focus	2	0	2	
PD-1 inhibitors	Tirilizumab	53	43	96	0.053
	Sindilizumab	41	39	80	
	Karelizumab	39	33	72	
	Teraplizumab	23	37	60	
	Navulizumab	19	33	52	
	Pembrolizumab	24	11	35	
	Srolizumab	1	4	5	

**Table 2:** Baseline characteristics of the total cohort and the two groups of patients.

Item	Sub-item	Total cohort (n=400)	Post-chemotherapy group (n=200)	Pre-chemotherapy group (n=200)	χ² value	P value
Sex	Male	254 (63.5%)	133 (66.5%)	121 (60.5%)	1.463	0.229
	Female	146 (36.5%)	67 (33.5%)	79 (39.5%)	–	–
Age (years)	Less than 30	12 (3.0%)	4 (2.0%)	8 (4.0%)	2.174	0.831
	30-39	38 (9.5%)	20 (10.0%)	18 (9.0%)	–	–
	40-49	80 (20.0%)	38 (19.0%)	42 (21.0%)	–	–
	50-59	118 (29.5%)	58 (29.0%)	60 (30.0%)	–	–
	60-69	92 (23.0%)	48 (24.0%)	44 (22.0%)	–	–
	70-79	56 (14.0%)	30 (15.0%)	26 (13.0%)	–	–
	More than 80	4 (1.0%)	2 (1.0%)	2 (1.0%)	–	–
Tumour type	Lung cancer	114 (28.5%)	86 (43.0%)	28 (14.0%)	64.521	<0.001
	Liver Cancer	60 (15.0%)	12 (6.0%)	48 (24.0%)	–	–
	Stomach cancer	42 (10.5%)	12 (6.0%)	30 (15.0%)	–	–
	Others	184 (46.0%)	84 (42.0%)	100 (50.0%)	–	–
PD-1 inhibitors	Tirilizumab	96 (24.0%)	53 (26.5%)	43 (21.5%)	12.476	0.053
	Sindilizumab	80 (20.0%)	41 (20.5%)	39 (19.5%)	–	–
	Karelizumab	72 (18.0%)	39 (19.5%)	33 (16.5%)	–	–
	Others	152 (38.0%)	67 (33.5%)	85 (42.5%)	–	–

### 3.2 Incidence of AEs in the two groups comparison

AEs occurred in 260 out of 400 patients, with an incidence rate of 65.0%. The incidence of AEs in the pre-chemotherapy group was 75.0% (150/200), which was significantly higher than that of 55.0% (110/200) in the second chemotherapy group, and the difference was statistically significant (P<0.05).

### 3.3 Occurrence of AEs comparison with the two groups

**Figure 1:**
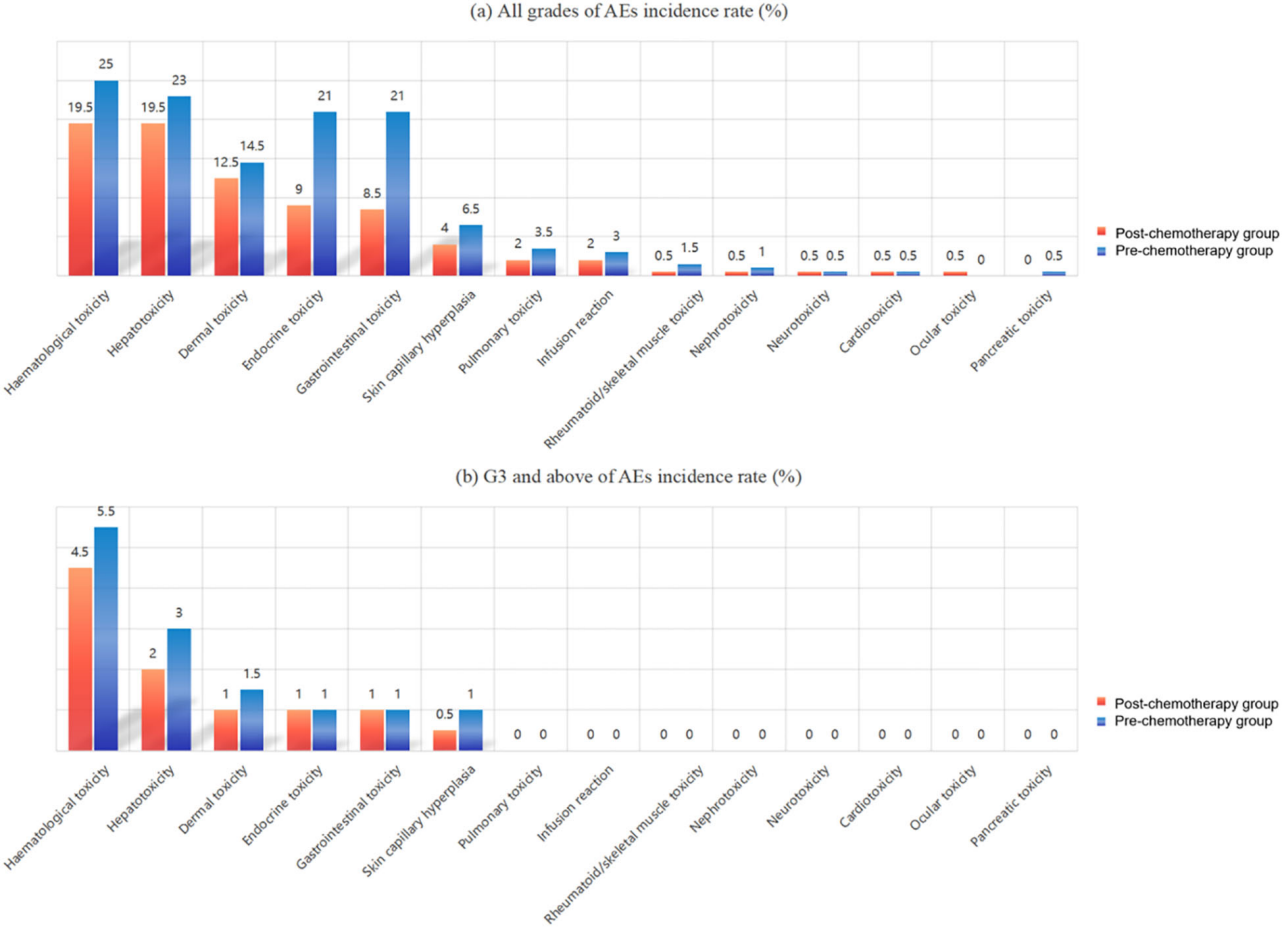
**(a)** All grades of adverse events incidence rate. **(b)** G3 and above of AEs incidence rate.

The types of AEs were more common in 89 cases (22.25%) of haematological toxicity, 85 cases (21.25%) of liver toxicity and 54 cases (13.5%) of skin toxicity. In the post-chemotherapy group, there were 139 patients with toxicity graded as G1~ G2 and 20 patients with G3 and above; in the first-chemotherapy group, there were 177 patients with G1~ G2 and 26 patients with G3 and above. There was no statistical difference in the incidence of similar AEs in either group. The types and grades of AEs occurring in the two groups are shown in **Table 3** and **Figure 1**.

**Table 3:** Types and grades of adverse events occurring in the two groups [n (%), n=200].

Type of AEs	Post-chemotherapy group	Pre-chemotherapy group	P value	Post-chemotherapy group	Pre-chemotherapy group	P value
	All levels	All grades		G3 and above	G3 and above	
Haematological toxicity	39 (19.5%)	50 (25%)	0.186	9 (4.5%)	11 (5.5%)	0.646
Hepatotoxicity	39 (19.5%)	46 (23%)	0.392	4 (2%)	6 (3%)	0.749
Dermal toxicity	25 (12.5%)	29 (14.5%)	0.558	2 (1%)	3 (1.5%)	1.000
Endocrine toxicity	18 (9%)	22 (21%)	0.505	2 (1%)	2 (1%)	1.000
Gastrointestinal toxicity	17 (8.5%)	22 (21%)	0.399	2 (1%)	2 (1%)	1.000
Skin capillary hyperplasia	8 (4%)	13 (6.5%)	0.262	1 (0.5%)	2 (1%)	1.000
Pulmonary toxicity	4 (2%)	7 (3.5%)	0.359	0 (0%)	0 (0%)	–
Infusion reaction	4 (2%)	6 (3%)	0.749	0 (0%)	0 (0%)	–
Rheumatoid/skeletal muscle toxicity	1 (0.5%)	3 (1.5%)	0.615	0 (0%)	0 (0%)	–
Nephrotoxicity	1 (0.5%)	2 (1%)	1.000	0 (0%)	0 (0%)	–
Neurotoxicity	1 (0.5%)	1 (0.5%)	1.000	0 (0%)	0 (0%)	–
Cardiotoxicity	1 (0.5%)	1 (0.5%)	1.000	0 (0%)	0 (0%)	–
Ocular toxicity	1 (0.5%)	0 (0%)	0.239	0 (0%)	0 (0%)	–
Pancreatic toxicity	0 (0%)	1 (0.5%)	0.239	0 (0%)	0 (0%)	–

The types of AEs included 14 types of skin toxicity, endocrine toxicity, cardiotoxicity, and malaise, and were classified into five grades based on toxicity: G1: mild toxicity; G2: moderate toxicity; G3: severe toxicity; G4: life-threatening toxicity; and G5: toxicity-related death, which corresponds to the Common Terminology Criteria for the Classification of Common Adverse Events (CTCAE_4.03), which was developed by the Cancer Research Institute of the National Institutes of Health (USA) for grading of adverse events.

Among the 260 patients who developed AEs, 176 patients developed only 1 type of AE, 66 patients developed 2 types of AEs, 18 patients developed 3 types of AEs, and no patient developed 4 or more types of AEs. The number of patients who developed ≥2 types of AEs in the post-chemotherapy group was 43, and the number of patients who developed ≥2 types of AEs in the first-chemotherapy group was 41.

### 3.4 Comparison of therapeutic effects between two groups

The focus of this study is to evaluate the impact of administration sequence on safety, therefore, data related to adverse events were mainly collected. Treatment evaluation indicators such as PFS, DFS, or OS were not included in the analysis scope of this study. However, all 400 patients in this study underwent radiographic examinations (CT and/or MR) to evaluate efficacy after 2 cycles of anti-tumour drug use, including 16 cases of CR (4%), 107 cases of PR (26.75%), 165 cases of SD (41.25%), and 112 cases of PD (28%).

The comparison between the two groups shows that their initial therapeutic effects are comparable. The ORR of the PD-1 inhibitor first administered and then chemotherapy group was 31.5% (63/200), with no statistically significant difference compared to the ORR of 30.0% (60/200) in the first chemotherapy group (P>0.05). Similarly, the DCR of the post chemotherapy group was 73.5% (147/200), while the DCR of the pre chemotherapy group was 70.5% (141/200), and there was no significant difference between the two groups (P>0.05). This discovery suggests that adopting a PD-1 inhibitor priority administration strategy may achieve the clinical optimisation goal of reducing drug toxicity without compromising efficacy.

## 4 Discussion

The present study shows that both groups of patients are predominantly male, and the age group is mostly concentrated in 40 ~ 69 years old, and the post-chemotherapy group is predominantly lung cancer patients, while the pre-chemotherapy group is predominantly hepatocellular carcinoma patients. The reason was considered to be the high incidence of lung and liver cancers in mainland China and the predominance of males. In addition, all seven PD-1 inhibitors included in this study are approved for non-small cell lung cancer and four are approved for hepatocellular carcinoma, which also explains why patients with lung cancer and hepatocellular carcinoma were mainly included in this study. The top three PD-1 inhibitors used in this study were tirilizumab, sindilizumab and karelizumab, and although pembrolizumab and navulizumab are the first two PD-1 inhibitors available in the world and in mainland China, the number of cases using these two PD-1 inhibitors in this study was relatively small, which is related to their high drug prices.

As new anti-tumour drugs, the mechanism of action of PD-1 inhibitors is different from that of traditional anti-tumour drugs, so the timing of their administration cannot be accurately grasped by the clinic, and there is a lack of relevant studies to support this. The results of this paper show that different dosing sequences will have an impact on the occurrence of AEs. The incidence of AEs in patients treated with PD-1 inhibitors followed by chemotherapy (55.0%) was much lower than that in patients treated with chemotherapy followed by PD-1 inhibitors (75.0%), and the difference was statistically significant (P < 0.05), but there was no statistically significant difference in the incidence of the same type of AEs in either group. It is also true that the current clinical preference is for the use of PD-1 inhibitors followed by chemotherapy, possibly in consideration of stimulating the body’s immune system first for maximum efficacy (9). The occurrence of AEs is associated with the over-activation of cellular immunity. In immunotherapy-combination chemotherapy regimens, chemotherapy can turn a “cold tumour” into a “hot tumour” that is sensitive to immunotherapy, and PD-1 inhibitors regulate the immune response induced by chemotherapy in a bi-directional manner, so that when patients are treated with chemotherapy and then PD-1 inhibitors, they may not be able to receive chemotherapy because of their body’s immune system being stimulated first. When patients are treated with PD-1 inhibitors after chemotherapy, the incidence of AEs may increase because of the oversensitive immune response (10–12).

The use of PD-1 inhibitors before chemotherapy may enhance the function of the immune system, improve the tumour microenvironment, and reduce the immune escape ability of tumour cells, thereby reducing the immune related adverse events caused by chemotherapy. PD-1 inhibitors activate the anti-tumour response of T cells by relieving immune checkpoint inhibition, which not only enhances immune clearance against tumours, but may also improve immune tolerance and reduce immune system overreaction. At the same time, PD-1 inhibitors can help repair immune suppression caused by chemotherapy, promote immune system recovery, and alleviate some side effects caused by chemotherapy, such as infection or inflammation. Overall, the use of PD-1 inhibitors before chemotherapy may improve patients’ treatment tolerance and reduce adverse events through multiple mechanisms.

The main types of AEs in the 400 patients included in this paper were haematological toxicity (22.25%), hepatotoxicity (21.25%) and dermatotoxicity (13.5%). Notably, when receiving PD-1 inhibitors in combination with chemotherapy, because some of the symptoms of chemotherapy-induced AEs and PD-1-induced AEs were the same, such as haematological toxicity and gastrointestinal toxicity, it was difficult for physicians to determine what drug-induced AEs were present and to attribute most of the occurrences of such AEs to chemotherapy. The overall incidence of haematological toxicity induced by PD-1 inhibitors was not high, less than 1%, and the most haematological toxicities are grade G2 or lower, with a small number of toxicities including haemolytic anaemia, aplastic anaemia, and immune thrombocytopenia, which can be life-threatening (13). The CheckMate 078 study showed that the incidence of anaemia among nabulizumab-associated haematological toxicities was approximately 4%, leukopenia was approximately 3%, neutropenia was approximately 2%, while all grade 3 to 4 toxicities were less than 1% (14). Phase I clinical studies of karelizumab showed an 11% incidence of anaemia, with 2% grade 3-4; 12% leukopenia and 1% thrombocytopenia, with no AEs of grade G3 or higher (15). A meta-analysis reported that PD-1 inhibitors caused 9.8% and 5% incidence of anaemia of any grade and G3 and above, respectively (16). Hematotoxicity in the present study was 22.25%, which is higher than the incidence of PD-1 inhibitor-induced hematotoxicity reported in most of the current literature, which is related to combination chemotherapy. The incidence of any grade of hepatotoxicity due to PD-1 inhibitors alone has been reported to be 0.7-2.1% (17), but the incidence of hepatotoxicity is much higher when combined therapy is used, such as in a meta-analysis of a Chinese population (18), where the incidence of any grade of hepatotoxicity was significantly higher after treatment with pembrolizumab, navulizumab, karelizumab, treprotilumab, tirilizumab, or sindilizumab After treatment, the incidence of any grade of liver toxicity ranged from 7.4 to 14.0%, with the incidence ranging from 6.9 to 13.1% for PD-1 inhibitor monotherapy and 12.2 to 37.8% for combination therapy, which is similar to the incidence of liver toxicity in this study. From the results of the available clinical studies, the incidence was about 34% to 40% in patients receiving nabulizumab and pabolizumab (19–21), but grade 3 to 4 rashes were rare. The incidence of pruritus was 13%-20% and 33% with PD-1 inhibitors and combination therapy, respectively, with a grade 3–4 incidence of <2.5% (22–24). The overall incidence of vitiligo is about 8% when PD-1 inhibitors are used in combination with ibritumomab (25, 26). The incidence of skin toxicity occurring in this study was slightly lower than that reported in the available literature, and the majority of skin toxicity was rash and pruritus, with a few cases of vitiligo.

The main types of AEs occurring in both groups were hematotoxicity, hepatotoxicity and dermatotoxicity. The mechanism of the occurrence of various types of AEs is still unclear, which requires close attention to the patient’s indicators, especially blood routine, during the treatment process. The toxicity grades of all types of AEs in both groups were concentrated in G1 to G2, and G3 and above were rare. There are five major points in the management of AEs, which are prevention, assessment, examination, treatment and monitoring, and prevention is the most important of these five links. This suggests that clinical attention should be paid to the occurrence of AEs and its importance, and that early identification and early intervention should be carried out to improve the outcome of patients by means of patient education and other means. PD-1 inhibitors administered before chemotherapy may reduce AEs, suggesting that dosing sequence should be considered in clinical practice.

Clinics are now dominated by immunotherapy combined with chemotherapy (27), and there is still an urgent need for relevant evidence-based medical evidence. Previous literature has mainly explored the safety of PD-1 inhibitors in combination with chemotherapy, and lacked an exploration of their dosing sequence.

In 2024, scholars studied the efficacy and safety of administering PD-1 inhibitors 3–5 days after TP chemotherapy in patients with locally advanced, recurrent, or metastatic oesophageal squamous cell carcinoma. The results showed that the administration sequence of PD-1 inhibitors 3–5 days after TP chemotherapy for first-line treatment of locally advanced, recurrent, or metastatic oesophageal squamous cell carcinoma not only achieved good efficacy, but also showed advantages in controlling specific adverse reactions. Under this sequence, the objective remission rate and disease control rate of patients are relatively high, and the incidence of grade 3–4 neutropenia and lymphopenia has decreased compared to previous reports. However, some patients still experience grade 3–4 toxicity events, with leukopenia accounting for the highest proportion. In addition, patients with a neutrophil to lymphocyte ratio of less than 3 before the third and fourth cycles of immunotherapy have a better prognosis (28).Zhang et al. explored the use of the PD-1 inhibitor tirelizumab in combination with albumin bound paclitaxel, followed by epirubicin/cyclophosphamide as a new adjuvant treatment for triple negative breast cancer in the Phase II TREND trial in 2025, but did not conduct a comparative analysis of the impact of different drug use sequences on adverse reactions. Under this medication regimen, the incidence of treatment-related adverse reactions is 90.57%, with common symptoms including hair loss, nausea, liver injury, etc. The incidence of grade 3–4 serious adverse reactions is only 11.32%, mainly liver injury, neutropenia, and leukopenia. The overall safety is controllable (29).

This study conducted a retrospective analysis of 400 cancer patients who received PD-1 inhibitor combined chemotherapy. The incidence of adverse reactions was compared between the group that received PD-1 inhibitor first and then chemotherapy (later chemotherapy group) and the group that received chemotherapy first and then PD-1 inhibitor (pre-chemotherapy group). It was found that the incidence of adverse reactions in the later chemotherapy group (55.0%) was significantly lower than that in the pre-chemotherapy group (75.0%). The main types of adverse reactions in both groups were haematological toxicity, hepatotoxicity, and skin toxicity, and there was no statistical difference in the incidence of similar adverse reactions. This result is expected to provide evidence-based support for the clear recommendation of the order of combination therapy in clinical drug guidelines, the standardisation of medication behaviour in actual treatment, and the optimisation of adverse reaction management. However, due to limitations such as limited sample size in the study, further large sample size is still needed. Prospective studies further validate.

The present study demonstrated that the dosing sequence would have an impact on the occurrence of AEs, which could provide ideas for clinical dosing regimens and the management of adverse effects. However, it should be noted that only 400 patients were included in this study, which has the problem of small sample size. There were also multiple limitations such as the failure to differentiate between adverse events caused by chemotherapeutic agents and those caused by PD-1 inhibitors, as well as the lack of consideration of effectiveness indicators. Therefore, more studies are needed to confirm the results of this study, and further exploration is needed to find out how the combination of PD-1 inhibitors with chemotherapy can be safer, more economical, more effective, and more reasonable, and we need to gain a deeper understanding of the mechanism of immunotherapy combined with chemotherapy, to further explore the optimal order of administration and the cycle and dosage of the combination of PD-1 inhibitors with chemotherapy, and to formulate a more efficient, less toxic, and more individualised treatment plan.

## Data Availability

The raw data supporting the conclusions of this article will be made available by the authors, without undue reservation.

## References

[1] Couzin-Frankel J . Cancer immunotherapy. Science. (2013) 342:1432–3. doi: 10.1126/science.342.6165.1432, PMID: 24357284

[2] Li Z Song W Rubinstein M Liu D . Recent updates in cancer immunotherapy: a comprehensive review and perspective of the 2018 China Cancer Immunotherapy Workshop in Beijing. J Hematol Oncol. (2018) 11:142. doi: 10.1186/s13045-018-0684-3, PMID: 30577797 PMC6303854

[3] Wang C Peng Y Chen F Zhang Z . Combination chemotherapy and order of medication in tumours. Chin Pharm. (2013) 24:2470–2.

[4] Xu Y Liu Y . Research progress of immune-related adverse events of PD-1 inhibitors. J Pract Oncol. (2020) 35:491–4.

[5] Man J Ritchie G Links M Lord S Lee CK . Treatment-related toxicities of immune checkpoint inhibitors in advanced cancers: a meta-analysis. Asia Pac J Clin Oncol. (2018) 14:141–52. doi: 10.1111/ajco.12838, PMID: 29349927

[6] Ministry of Health of the People’s Republic of China . Administrative measures for reporting and monitoring of adverse drug reaction. Available online at: http://www.gov.cn/flfg/2011-05/24/content_1870110.htm.

[7] Zhang L Li b Li R Ding C . Toxicities and management of immune checkpoint inhibitors in cancer immunotherapy. Int Respir J. (2019) 39(04):312–6. doi: 10.3760/cma.j.issn.1673-436X.2019.04.012.

[8] Guidelines Working Committee of the Chinese Society of Clinical Oncology . Chinese Society of Clinical Oncology (CSCO) guidelines for the management of toxicity associated with immune checkpoint inhibitors. Beijing: People’s Health Publishing House (2021) p. 01–148.

[9] Qiu J Lu C Zeng X Chen Z Liu W Wei T . A preliminary discussion on PD-1 inhibitor combination regimen and dosing sequence in ambulatory chemotherapy centres. Pharm Today. (2019) 29:812–5. doi: 10.12048/j.issn.1674-229X.2019.12.004

[10] Duan Q Zhang H Zheng J Zhang L . Turning cold into hot:firing up the tumour microenvironment. Trends Cancer. (2020) 6:605–18. doi: 10.1016/j.trecan.2020.02.022, PMID: 32610070

[11] Pozzi C Cuomo A Spadoni I Magni E Silvola A Conte A . The EGFR-specific antibody cetuximab combined with chemotherapy triggers immunogenic cell death. Nat Med. (2016) 22:624 –631. doi: 10.1038/nm.4078, PMID: 27135741

[12] Hao S He Y . From immunological monotherapy to multidrug combination - the preferred path of PD-1 inhibitors for NSCLC. Chongqing Med. (2018) 47:2885–2888+2904. doi: 10.3969/j.issn.1671-8348.2018.22.001

[13] Delanoy N Michot JM Comont T Kramkimel N Lazarovici J Dupont R . Haematological immune-related adverse events induced by anti-PD-1 or anti-PD-L1 immunotherapy: a descriptive observational study. Lancet Haematol. (2019) 6:e48–57. doi: 10.1016/S2352-3026(18)30175-3, PMID: 30528137

[14] Wu YL Lu S Cheng Y Zhou C Wang J Mok T . Nivolumab versus docetaxel in a predominantly chinese patient population with previ.ously treated advanced NSCLC. CheckMate 078 randomized phase llclinical trial. J Thorac Oncol. (2019) 14:867–75. doi: 10.1016/j.jtho.2019.01.006, PMID: 30659987

[15] Fang W Yang Y Ma Y Hong S Lin L He X . Camrelizumab (SHR-1210) alone or in combination with gemcitabine plus cisplatin for nasopharyngeal carcinoma: Results from two single-arm, phase 1 trials. Lancet Oncol. (2018) 19:1338–50. doi: 10.1016/S1470-2045(18)30495-9, PMID: 30213452

[16] Petrelli F Ardito R Borgonovo K Lonati V Cabiddu M Ghilardi M . Haematological toxicities with immunotherapy in patients with cancer: a systematic review and meta- analysis. Eur J Cancer. (2018) 103:7–16. doi: 10.1016/j.ejca.2018.07.129, PMID: 30196108

[17] Peeraphatdit TB Wang J Odenwald MA Hu S Hart J Charlton MR . Hepatotoxicity from immune checkpoint inhibitors: a systematic review and management recommendation. Hepatology. (2020) 72:315–29. doi: 10.1002/hep.31227, PMID: 32167613

[18] Li L Li G Rao B Dong AH Liang W Zhu JX . Landscape of immune checkpoint inhibitor-related adverse events in Chinese population. Sci Rep. (2020) 10:15567. doi: 10.1038/s41598-020-72649-5, PMID: 32968172 PMC7511303

[19] Haanen J Obeid M Spain L Carbonnel F Wang Y Robert C . Management of toxicities from immunotherapy: ESMO ClinicalPractice Gjuidelines for diagnosis, treatment and follow-up. Ann Oncol. (2017) 28:iv119–42. doi: 10.1093/annonc/mdx225, PMID: 28881921

[20] Hodi FS O'Day SJ McDermott DF Weber RW Sosman JA Haanen JB . Improved survival with ipilimumab in patients with metastatic melanoma. N Engl J Med. (2010) 363:711–23. doi: 10.1056/NEJMoa1003466, PMID: 20525992 PMC3549297

[21] Robert C Schachter J Long GV Arance A Grob JJ Mortier L . Pembrolizumab versus ipilimumab in advanced melanoma. N Engl J Med. (2015) 372:2521–32. doi: 10.1056/NEJMoa1503093, PMID: 25891173

[22] Puzanov I Diab A Abdallah K Bingham CO Brogdon C Dadu R . Managing toxicities associated with immune checkpoint inhibitors: consensus recommendations from the Society for lmmunotherapy of Cancer (SITC) Toxicity Management Working Group. J Immunother Cancer. (2017) 5:95. doi: 10.1186/s40425-017-0300-z, PMID: 29162153 PMC5697162

[23] Larkin J Chiarion-Sileni V Gonzalez R Grob JJ Cowey CL Lao CD . Combined nivolumab and ipilimumab or monotherapy in untreated melanoma. N Engl J Med. (2015) 373:23–34. doi: 10.1056/NEJMoa1504030, PMID: 26027431 PMC5698905

[24] Weber JS Hodi FS Wolchok JD Topalian SL Schadendorf D Larkin . Safety profile of nivolumab monotherapy: a pooled analysis of patients with advanced melanoma. J Clinl Oncol. (2017) 35:785–92. doi: 10.1200/JCO.2015.66.1389, PMID: 28068177

[25] Hofmann L Forschner A Loquai C Goldinger SM Zimmer L Ugurel S . Cutaneous, gastrointestinal, hepatic, endocrine, and renal side-effects of anti-PD-1 therapy. Eur J Cancer. (2016) 60:190–209. doi: 10.1016/j.ejca.2016.02.025, PMID: 27085692

[26] Hua C Boussemart L Mateus C Routier E Boutros C Cazenave H . Association of vitiligo with tumour response in patients with metastatic melanoma treated with pembrolizumab. JAMA Dermatol. (2016) 152:45–51. doi: 10.1001/jamadermatol.2015.2707, PMID: 26501224

[27] Tang HC Meng J Xia ZZ Lian Z Chen Y . A retrospective study of the safety of immune checkpoint inhibitors in the treatment of advanced non-small cell lung cancer. Pharm Today. (2025) 35(6):457–62. Online First. doi: 10.12048/j.issn.1674-229X.2025.06.009

[28] Shen L Chen ZX Zhang Z Wu YJ Ren Y Li Y . Clinical efficacy of taxol plus platinum (TP) chemotherapy combined with delayed administration of PD-1 inhibitors in patients with locally advanced, recurrent or metastatic esophageal squamous cell carcinoma: A retrospective study. Drug Des Dev Ther. (2024) 18:2761–73. doi: 10.2147/DDDT.S455248, PMID: 38979399 PMC11230125

[29] Zhang Q Wang M Li Y Zhang H Wang Y Chen X . Efficacy, safety and exploratory analysis of neoadjuvant tislelizumab (a PD-1 inhibitor) plus nab-paclitaxel followed by epirubicin/cyclophosphamide for triple-negative breast cancer: A phase 2 TREND trial. Signal Transduct Target Ther. (2025) 10:169. doi: 10.1038/s41392-025-02254-3, PMID: 40414961 PMC12104340

